# Association of Screen Time with Sleep Duration in School-Aged Children; a Nationwide Propensity Score‑Matched Analysis: The CASPIAN‑V Study

**Published:** 2019-05-29

**Authors:** Shahrzad Mortazavi, Mohammadesmaeil Motlagh, Mostafa Qorbani, Nafiseh Mozafarian, Ramin Heshmat, Roya Kelishadi

**Affiliations:** ^1^Department of Mental Health, Child Growth and Development Research Center, Research Institute for Primordial Prevention of Non‑communicable Disease, Isfahan University of Medical Sciences, Isfahan, Iran; ^2^Department of Pediatrics, Ahvaz Jundishapur University of Medical Sciences, Ahvaz, Iran; ^3^Non-communicable Diseases Research Center, Alborz University of Medical Sciences, Karaj, Iran; ^4^Endocrinology and Metabolism Research Center, Endocrinology and Metabolism Clinical Sciences Institute, Tehran University of Medical Sciences, Tehran, Iran; ^5^Chronic Diseases Research Center, Endocrinology and Metabolism Population Sciences Institute, Tehran University of Medical Sciences, Tehran, Iran; ^6^Department of Pediatrics, Child Growth and Development Research Center, Research Institute for Primordial Prevention of Non‑communicable Disease, Isfahan University of Medical Sciences, Isfahan, Iran

**Keywords:** Children, Adolescents, Propensity score, Screen time, Sleep duration

## Abstract

**Background:** This study aims to determine the association of sleep duration with screen time in children and adolescents.

**Study design:** A matched case-control study.

**Methods:** This nationwide study was conducted in 2015 among 14,274 students aged 7-18 years, and one of their parents who lived in 30 provinces in Iran. Data collection was performed using questionnaires and physical examination. Watching television and working with computer were categorized into two groups (<2 h/day and ≥2 h). Moreover short sleep duration was defined as sleep duration ≤ 8 h/day. The analysis was conducted based on the propensity score using a matched case– control study design. Data analysis was performed by a conditional logistic regression.

**Results:** Overall, 14,274 students and one of their parents completed the survey (participation rate: 99%). Mean (standard deviation) age of students was 12.3 (3.2) years, and the frequency of short sleep was (4672) 33.5% .In total, 54.3% of students watched TV ≥2 h/day and 9% of those used a computer ≥2 h/day in their leisure time. In the multivariate model, Individuals who watched TV ≥2 h/day had significantly higher odds of short sleep (OR 1.13, 95% CI 1.03, 1.24), and individuals who worked with computer ≥2 h/day had significantly higher odds of short sleep (OR 1.65, 95% CI 1.40, 1.94).

** Conclusion:** This study revealed significant association between watching TV and using computer during leisure time with insufficient sleep. Public awareness should be provided regarding the adverse effects of screen use on sleep. Developing special guide lines and educational school programs on restricting screen time and increasing physical activities should be considered as a health priority for children and adolescents.

## Introduction

Sleep is a biological and psychological necessity. Adequate sleeping, especially in childhood and adolescence have an important influence on cognitive abilities, mood and performance^1^.

Sleep problems are common among school ages. It is estimated that approximately 25 percent of children will suffer from a sleep problem at some point during childhood. About 30 percent of school-aged children and 40 percent of adolescents have sleep difficulties^2^.

Sleep loss has multiple sequels on cognitive and emotional states of children and adolescents. Evidences show that inadequate sleep in childhood leading to attention disturbance, poor decision-making and decreased memory, which may contribute to a poorer academic success ^3, 4^ in addition to increased risk of obesity, insulin resistance and hypertension^5-7^. Mental effects of sleep disturbance among adolescents include depression, suicidal ideation and emotional deregulation^8-10^.

Sleep demands change across different ages. Even though there is inter individual variations, the latest recommendations of the American Academy of Sleep Medicine state that the appropriate sleep duration among 6 to 12 years old is 9 to 12 hours and in teenagers, between 13 to 18 years, is 8 to 10 hours per day^11^.

Sleep patterns change during life time. In adolescence, from age 10 to 19 years, sleep changes may accrue due to verity of internal and external factors. Beside changes in biological clock and social obligations and academic demands, availability of television, video games, computer and telephone alter the sleep patterns in adolescents^12^.

Studies have shown negative effects of television on physical and behavioral developments of children and adolescents. Sleep disturbance manifest as irregular sleep habits, shorter sleep duration, and sleep disorders .Use of computer and mobile phones correlates with interrupted sleep and sleep difficulties^13^.

In recent years we have witnessed a rapid increase in the availability and use of electronic devices such as television and portable electronic devices. According to Iran statistics center, more than 98 percent of Iranian families have a television and about 97 percent of them own a phone or cell phone and the number of personal computers is about 10.5 million. These changes have made a great concern of rapidly growing use of screens on health issues, especially among school-aged and adolescent. This study aims to detect the association between sleep duration and screen time in a large sample of pediatric population.

## Methods

### Study population

Data of this matched case-control study were obtained from the fifth phase of a national project entitled “Childhood and Adolescence Surveillance and Prevention of Adult Non-communicable disease” (2014–2015). The Protocol details of the CASPIAN-V study have been described previously ^2^, and here we present it in brief.

This national research was conducted as a Multicenter cross sectional study on 7–18 year old students with the Cooperation and coordination of the Ministry of Health and Medical Education, Ministry of Education, Child Development Research Center (CDRC) at the Isfahan University of Medical Sciences, and Endocrinology and Metabolism Research Institute (EMRI) at the Tehran University of Medical Sciences. The protocol of study was confirmed by the Ethics Committee of Isfahan University of Medical Sciences .Verbal and written consent was obtained from the students and their parents, respectively. The students were randomly selected from urban and rural areas in 30 provinces of Iran (48 clusters of 10 people in each province) by a stratified, multi-stage cluster sampling design. In total 14,400 people were surveyed in 30 provinces of the country.

### Data collection

Data were collected using a structured questionnaire, clinical examinations and anthropometric measurements .The internal consistency of the questionnaire was Confirmed in the previous studies of Caspian with the overall Cranach's alpha value of 0.93 and range of 0.92 to 0.97, and the reliability was approved as 0.94 by test–retest measurement ^15^. Students’ demographic characteristics, healthy behaviors (physical activity, time spent watching TV, using computers, sleep duration and homework) were asked through students’ questionnaires and some complementary data on family characteristics, number of children in the family, birth order, parental age and socioeconomic variables questioned through parents’ questionnaires.

Also, a number of questions about some mental disorders (1- During the past 12 months, did you ever feel sad or hopeless so that you cannot do your daily activity? 2-During the past 6 months, did you experience Early anger and irritability? 3-During the past 6 months, did you experience anxiety?) were asked from the students.

### Socioeconomic status

The socioeconomic status (SES) score was calculated using the Principle component analysis method based on parents’ education, parents’ job, type of school (private or public), type of house, having personal computer in house and possessing private car. Provided measure was categorized as low, medium and high socioeconomic status.

Physical activity (hours/day) was estimated using a validated questionnaire. Students were asked about 7-day recalls of sports or activities in and out of school activities that led to sweating and increasing in breathing or heart rate. All physical activity items were summarized in a main variable through principal component analysis (PCA) methods. This main variable was categorized into tertiles. The first tertile was defined as a low, second tertile as a medium and third tertile as a high PA ^16^.

### Sedentary behaviors

The time spent watching TV and using computers during leisure time was assessed by a questionnaire. The students were asked to report number of hours per day spent on watching TV, PC and electronic games. Watching television and working with computer were categorized into two groups (<2 h/day: 0 and ≥2 h/day:1) ^17^.

Moreover, students reported that they usually sleep several hours on weekdays and on weekends. For each student the weighted average hours of sleeping per day was calculated. Sleep duration was then considered as a dichotomous variable. Short sleep duration was defined as sleep duration ≤ 8 h/day^18^. (Long sleepers: 0, short sleepers: 1)

### Anthropometric measurements

A trained team of health care providers measured the weight and height of students under standard protocols using calibrated instruments ^15^. Height was measured without shoes to the nearest 0.1 cm using a non-elastic tape meter and Students’ weights were measured on a scale placed on a flat ground to the nearest 0.1 kg. BMI was calculated as weight (kg) divided by square of height (m^2^).

### Statistical analysis and matching based on Propensity Score

In order to describe and initial analysis quantitative and qualitative variables based on short sleep, independent t-test, Chi-square test were used, respectively. The quantitative variables are reported as mean and standard deviation (SD) and the qualitative variables as frequency and percentage.

Propensity Score (PS) was calculated based on conditional logistic model with potential Confounding variables (age, sex, living area, SES, PA, homework, BMI, Birth order, number of children in the family , parental age and Some mental disorders (During the past 12 months, did you ever feel sad or hopeless so that you cannot do your daily activity? , During the past 6 months, did you experience Early anger and irritability? , During the past 6 months, did you experience anxiety ? ).Two groups (short sleepers and long sleepers) were matched based on 1:1 matching method without replacement by the score. Results of conditional logistic regression were reported as odds ratios (OR) and 95% confidence intervals (CI).

In addition, we have also implemented Logistic regression analysis. STATA- 10 (Stata Corp, College Station, Texas, USA) was used to analyze the data set. The P- value less than 0.05 were considered as significant.

## Results

Overall, 14274 students (50/6% boys) and (71.4%) urban inhabitant from 14400 invited students (participation rate: 91.5%) were participated in the current study. Participants consisted of 14274 students, 7228 (50/6%) boys and 10194 (71.4%) urban inhabitant. The mean (SD) age of the participants was 12.3(3.2) years.

The mean (SD) sleep duration of students was 8.57(1.23) hours in day and the frequency of short sleep was(4672 )33.5% (sleep duration ≤ 8 h/day).In total, 54.3% of students watched TV ≥2 h/day, 9% of those used a computer ≥2 h/day in their leisure time.

The PS was calculated according to conditional logistic model with potential confounders. Short sleepers and long sleepers were matched using one to one PS matching method. Table 1 shows, the distribution of characteristics of subjects in both groups prior to and after matching.

**Table 1:** Characteristics of the study participants according to daily sleep duration: The CASPIAN-V Study

**Sleep duration**	**Before Propensity Score- Matched**					**After Propensity Score- Matched**				
	**Long >8 h** **n = 9270**		**Short ≤8 h** **n=4672**		***P*** **value**	**Long >8 h** **n =3783**		**Short ≤8 h** **n =3783**		***P *** **value**
**Continuous variables**	**Mean**	**SD**	**Mean**	**SD**		**Mean**	**SD**	**Mean**	**SD**	
Age (yr)	12.14	3.12	12.57	3.12	0.001	12.61	3.17	12.62	3.22	0.905
Body mass index (kg/m^2^)	18.41	4.17	18.70	4.72	0.001	18.78	5.35	18.75	4.80	0.786
Parental age (w)	39.00	7.04	39.60	6.90	0.001	39.70	7.08	39.60	6.90	0.440
Number of children in the family	3.09	1.58	3.20	1.63	0.012	3.16	1.59	3.14	1.63	0.689
Birth order	2.17	1.52	2.17	1.49	0.972	2.18	1.49	2.16	1.51	0.592
**Continuous variables**	**Number**	**Percent**	**Number**	**Percent**		**Number**	**Percent**	**Number**	**Percent**	
Sex					0.594					0.713
Boy	4712	50.9	2353	50.4		1925	50.9	1909	50.5	
Girl	4552	49.1	2317	49.6		1858	49.1	1874	49.5	
Socioeconomic status					0.012					0.708
Low	2942	33.2	1509	33.8		1172	31.0	1201	31.8	
Moderate	3015	34.0	1405	31.5		1268	33.5	1239	32.7	
high	2914	32.8	1546	34.7		1343	35.5	1343	35.5	
Living area					0.001					0.814
Urban	6510	70.2	3424	73.3		2797	73.9	2788	73.7	
Rural	2760	29.8	1248	26.7		986	26.1	995	26.3	
Physical activity					0.001					0.101
Low	2594	29.5	1805	42.5		1502	39.7	1570	51.5	
Moderate	3024	34.4	1309	30.8		1150	30.4	1163	30.7	
high	3177	36.1	1135	26.7		1131	29.9	1050	27.8	
Depression					0.001					0.522
No	6506	70.7	2770	60.0		2272	60.1	2225	58.8	
Yes	1352	14.7	1096	23.8		856	22.6	891	23.6	
I do not remember	1342	14.6	748	16.2		655	17.3	667	17.6	
Aggression					0.001					0.927
No	6136	66.7	2443	53.3		1881	49.7	1885	49.8	
yes	3057	33.2	2145	46.7		1902	50.3	1898	50.2	
Anxiety					0.736					0.751
No	6148	67.3	3125	67.0		2512	66.4	2525	66.7	
yes	2987	32.7	1538	33.0		1271	33.6	1258	33.3	
Homework					0.001					0.579
<2h	3886	42.0	1278	27.4		1122	29.7	1100	29.1	
≥2h	5357	58.0	3382	72.6		2661	70.3	2683	70.9	

The total number of participants in the pre-matching sample was 13942 (long sleepers: 9270, short sleepers: 4672). In contrast, the total number of participants in the post-matching sample was 7566 (long sleepers: 3783, short sleepers: 3783).

**Figure 1:**
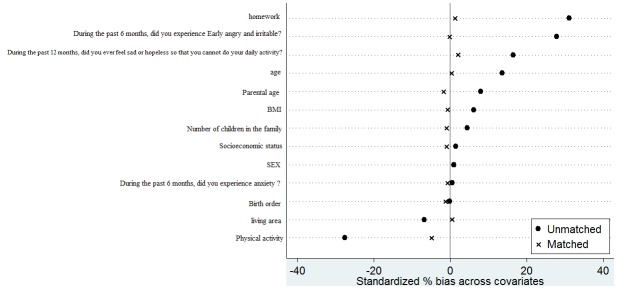
Standardized differences for general characteristics in the original and the matched sample: The CASPIAN-V Study

The mean of Standardized Bias percentages before and after matching were calculated. After matching, the mean of Standardized Bias percentage was reduced from 10.3 to 1.2%which shows the high quality of the matching and ensures that the distribution of the variables was not statistically significant between two groups (Figure 1).

Results of multivariate conditional logistic regression for association between watching TV and working computer with short sleep are presented in Table 2.Individuals who watched TV ≥2 h/day had significantly higher odds of short sleep (OR 1.13, 95% CI 1.03, 1.24). Individuals who worked computer ≥2 h/day had significantly higher odds of short sleep (OR 1.65, 95% CI 1.40, 1.94).

**Table 2:** Results of the Conditional logistic of watching TV and computer use in relation with sleep duration: The CASPIAN-V Study

	**Unadjusted Logistic regression**		**Adjusted Logistic regression** ^a^		**Matched Propensity Score**	
	**OR (95% CI)**	***P*** **value**	**OR (95% CI)** ^a^	***P*** **value**	**OR (95% CI)**	***P*** **value**
**Watching TV**	1.14 (1.06, 1.22)	0.001	1.09 (1,00, 1.18)	0.050	1.13 (1.03, 1.24)	0.010
**Computer use**	1.41 (1.25, 1.59)	0.001	1.73 (1.51, 1.98)	0.001	1.65 (1.40, 1.94)	0.001

^a^ Adjusted for all variables presented in Table 1

In addition, Multiple Logistic regression analysis after controlling the variables such (age, sex, living area, SES, PA, homework, BMI, Birth order, number of children in the family, parental age and Some mental disorders (During the past 12 months, did you ever feel sad or hopeless so that you cannot do your daily activity?, During the past 6 months, did you experience early anger and irritability?, During the past 6 months, did you experience anxiety? ) showed that individuals who watched TV ≥2 h/day had significantly higher odds of short sleep (OR 1.09, 95% CI 1.00, 1.18).Findings of this study also showed that individuals who worked computer ≥2 h/day had significantly higher odds of short sleep (OR 1.73, 95% CI 1.51, 1.98).

## Discussion

The main objective of this study was to assess the relationship between screen-time and sleep parameters such as sleep duration among school-aged and adolescents. Findings showed that watching TV or working with computer more than 2 h/day was associated with short sleep duration. Utilization of electronic devices is prevalent during adolescence and previous studies revealed the negative association of using TV with sleep duration in addition to delay in bed time and wake up time among adolescents^21^. Over use of computer would lead to sleep problems and bed time reduction ^3, 24^. In conclusion, using electronic media has been related to sleep reduction and bed time delay, based on a review of literature ^25^.

Electronic devices could affect sleep through different mechanisms. Time consuming nature of media use may interfere with normal sleep schedule^26^. Unstructured activities can displace other unstructured activities such as sleep. Because media use usually peaks before bedtime, sleep would be displaced by the media^27^.

As a result of availability of various forms of electronic media, children and adolescents are being engaged in sedentary activities which defined as sitting or reclining posture and low-energy expenditure. This can increase the risk of sleep disturbance and insomnia^28^. Obesity as a result of sedentary life style leads to sleep disturbances such as shorter duration of sleep and obese individuals present higher levels of chronic emotional stress than non-obese subjects^29^.

Bright light exposure which is the consequence of electronic media devices may interfere with sleep by inducing misalignment between the sleep–wake cycle and the internal clock ^25^. It has been found that Light exposure in the evening, increase alertness and arousal levels, suppress melatonin production^30^ and induce phase delay in the circadian clock such as delay in sleep time^31^.

Study limitations and strengths: This study is a cross sectional study and we cannot confirm the casual relationship between screen time and sleep difficulties. Data were based on self-reports so recall biases may interfere with results. Other variables such as psychological status and physical activities that have possible effects on sleep were not analyzed. The main strength of this study is the large sample size and to our knowledge it is the largest study of screen time relationship with sleep deficits in pediatric population and participants were gathered from different parts of Iran and included various socio demographic features. Moreover the matching method based on propensity score was used as the method for controlling a large number of probably confounding variables. Publication of results recently based on this method has been increased dramatically^32^.This method reduced the possibility of confounding and made the results more reliable.

## Conclusion

According to this study there is a strong relationship between screen time and sleep difficulties. Because of importance of adequate sleep from one hand and high rate of screen use on the other hand, policy makers and individuals should be informed to limit the screen time. Special guide lines for healthy use of screens should be developed according to different ages. School programs have an important role in promoting health issues and are recommended for students and parents to give them more understanding regarding the use of television and computer. Parents have to monitor and control leisure time activities of their children and consider more active and creative ways to spend time together.

## Acknowledgements

The authors are thankful of all participants and large team working on this project in different provinces.

## Conflict of interest

The authors declare no conflicts of interest.

## Funding

The study was conducted as a national school-based surveillance program.This work was supported by Child Department of Pediatrics, Child Growth and Development Research Center, Research Institute for Primordial Prevention of Non-communicable Disease, Isfahan University of Medical Sciences, Isfahan, Iran. The funders had no role in study design, data collection and analysis, decision to publish, or preparation of the manuscript.

## Highlights

- Significant association was found between screen times and sleep duration.
- Watching TV more than 2 h/day was associated with short sleep duration.
- Working with computer more than 2 h/day was associated with short sleep duration.
